# Supplementary figures and images for: Targeting host deoxycytidine kinase mitigates Staphylococcus aureus abscess formation
Source: eLife. 2024 Mar 21;12:RP91157. doi: 10.7554/eLife.91157 (PMC10957174; doi:10.7554/eLife.91157)

Figure 3E

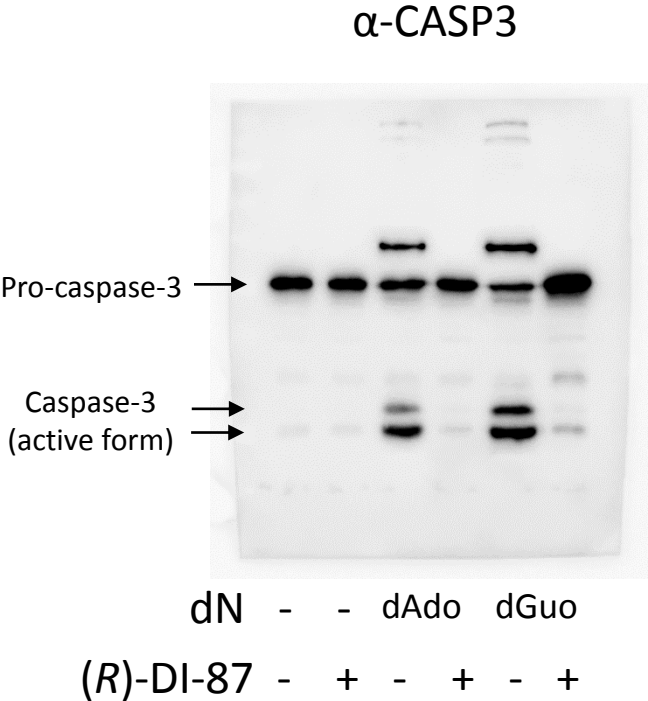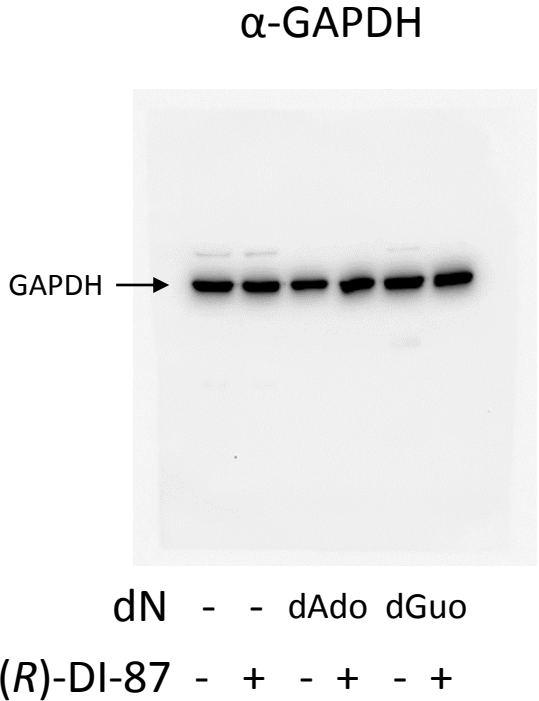

Supplement: Figure 3—source data 2. [file elife-91157-fig3-data2.zip › Immunoblots Figure 3E - labeled.pdf]

Figure 3F

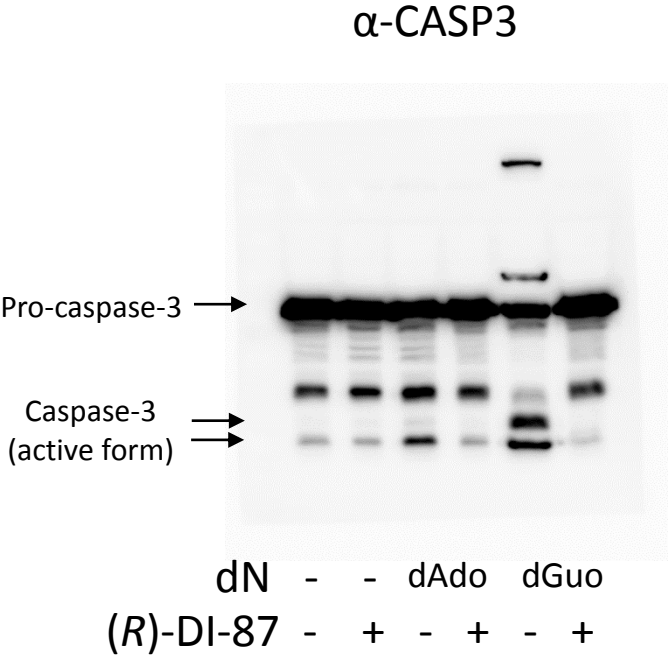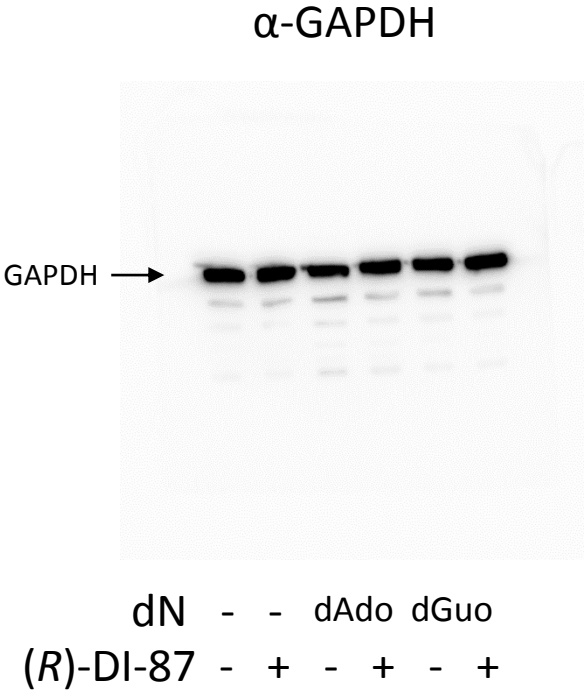

Supplement: Figure 3—source data 2. [file elife-91157-fig3-data2.zip › Immunoblots Figure 3F - labeled.pdf]
