# Supplementary material for: Targeting host deoxycytidine kinase mitigates Staphylococcus aureus abscess formation
Source: eLife. 2024 Mar 21;12:RP91157. doi: 10.7554/eLife.91157 (PMC10957174; doi:10.7554/eLife.91157)
Supplement: Supplementary file 1. [file elife-91157-supp1.docx]

**Supplementary file 1.** Minimum inhibitory concentration of (*R*)-DI-87

| **Bacterial strain** | **MIC (µg/ml)** |
| --- | --- |
|  | **(*R*)-DI-87** |
| *S. aureus* Newman wild type | > 2048 |
| *S. aureus* Newman ∆*adsA* | > 2048 |
